# Risk assessment in precapillary pulmonary hypertension: a comparative analysis

**DOI:** 10.1186/s12931-021-01624-z

**Published:** 2021-01-21

**Authors:** Thomas Sonnweber, Eva-Maria Schneider, Manfred Nairz, Igor Theurl, Günter Weiss, Piotr Tymoszuk, Judith Löffler-Ragg

**Affiliations:** Department of Internal Medicine II, Medical University Innsbruck, Anichstraße 35, 6020 Innsbruck, Tyrol Austria

**Keywords:** Pulmonary hypertension, Biomarkers, Risk assessment, Cardiovascular disease, Mortality

## Abstract

**Background:**

Risk stratification is essential to assess mortality risk and guide treatment in patients with precapillary pulmonary hypertension (PH). We herein compared the accuracy of different currently used PH risk stratification tools and evaluated the significance of particular risk parameters.

**Methods:**

We conducted a retrospective longitudinal observational cohort study evaluating seven different risk assessment approaches according to the current PH guidelines. A comprehensive assessment including multi-parametric risk stratification was performed at baseline and 4 yearly follow-up time-points. Multi-step Cox hazard analysis was used to analyse and refine risk prediction.

**Results:**

Various available risk models effectively predicted mortality in patients with precapillary pulmonary hypertension. Right-heart catheter parameters were not essential for risk prediction. Contrary, non-invasive follow-up re-evaluations significantly improved the accuracy of risk estimations. A lack of accuracy of various risk models was found in the intermediate- and high-risk classes. For these patients, an additional evaluation step including assessment of age and right atrium area improved risk prediction significantly.

**Discussion:**

Currently used abbreviated versions of the ESC/ERS risk assessment tool, as well as the REVEAL 2.0 and REVEAL Lite 2 based risk stratification, lack accuracy to predict mortality in intermediate- and high-risk precapillary pulmonary hypertension patients. An expanded non-invasive evaluation improves mortality risk prediction in these individuals.

## Background

Pulmonary hypertension (PH) affects 1% of the global population and is mainly related to heart and lung diseases [1]. Current ESC/ERS guidelines define PH as a mean pulmonary arterial pressure (mPAP) ≥ 25 mmHg as measured via right heart catheterization (RHC) at rest, and according to the etiology, five different WHO groups are defined [2]. Additionally, hemodynamic parameters define precapillary, postcapillary, and combined pre/postcapillary forms of PH [2]. These classifications are of high clinical relevance, as various subgroups of PH vastly differ in their pathobiology and prognosis, and urge for differential treatment approaches.

Current guidelines and expert reports recommend repetitive multi-parametric risk assessment in patients with WHO group I PH, also referred to as pulmonary arterial hypertension (PAH), and various differential tools have been evaluated for this purpose [2–14]. Although, currently available risk stratification models rely on similar variables and cut-offs, they vastly vary in the number of included parameters, ranging from 3 to 14, the number of defined risk classes, and the mode of risk class calculation. Multiple risk parameters have been identified for PH including the etiology of PH, RHC derived parameters [e.g. pulmonary vascular resistance, pulmonary arterial pressure (PAP) and right atrial pressure (RAP), cardiac index (CI), mixed venous oxygen saturation (SvO_2_)], performance status [e.g. 6-min walking distance (SMWD), WHO functional class (WHOFc), and peak oxygen consumption (VO_2_ peak) assessed in cardiopulmonary exercise testing], markers acquired with echocardiography [e.g. right atrial area (RAA) and the presence of pericardial effusion] and patient characteristics such as age and male gender [15–17]. Additionally, glomerular filtration rate (GFR), brain-natriuretic peptide (BNP), and N-terminal pro-natriuretic peptide (Nt-proBNP) are established laboratory biomarkers for PH.

To date, the ESC/ERS risk table and the US Registry to Evaluate Early and Long-term PAH Disease Management (REVEAL) score are most commonly used in clinical practice. The ESC/ERS risk assessment tool includes only modifiable risk parameters and was validated in abbreviated versions by the French Pulmonary Hypertension Registry (FPHR), the German Prospective Registry of Newly Initiated Therapies for Pulmonary Hypertension (COMPERA), and the Swedish PAH register (SPAHR). COMPERA and SPAHR define three cut-offs for each included parameter, representing a low-, intermediate- and high-risk strata [4, 9]. These strata are converted into numeric values (e.g. 1 for low-risk, 2 for intermediate-risk, and 3 for high-risk) and used to calculate the average risk category for a given patient (obtained by the addition of risk scores for each parameter and division by the number of used parameters). In contrast, the FPHR model uses four parameters to define low-risk criteria and stratifies mortality risk according to the number of criteria met [7]. The original REVEAL risk model and its updated version REVEAL 2.0, rate mortality risk according to the presence of various modifiable and non-modifiable patient characteristics as well as clinical, functional, exercise, laboratory, and hemodynamic parameters [6, 16, 18]. Each category is rated with a weighted score, and the score for each category is added to obtain a total sum score, which defines five different risk classes (low, intermediate low, intermediate, intermediate high, and very high, respectively). In contrast to the complex multi-parametric ESC/ERS and REVEAL models, there have been attempts to develop simple risk assessment tools, which rely on only a few parameters and do not depend on measurements from right heart catheterization (RHC). In this context, some centres endorse the use of the three-parametric FPHR score (FPHR3p) or the modified Risk Assessment Score of PAH (mRASP) [7, 8].

The existence of multiple different well-established approaches for risk stratification in PH offers the clinician the convenience of choice but also results in uncertainties and pitfalls, and hinders the establishment of a generally usable and comparable tool to assess mortality risk in PH patients. Thus, there is an ongoing discussion on how to harmonize and standardize risk assessment across various PH centers. In this context, we herein evaluate seven different risk assessment tools in a cohort of precapillary PH patients, explore the role of baseline and follow-up risk assessment, and offer a novel approach to refine currently available risk stratification models.

## Methods

### Study population and design

We herein performed a retrospective longitudinal observational cohort study. 153 patients with precapillary pulmonary hypertension according to RHC evaluation (mPAP ≥ 25 mmHg, PCWP ≤ 15 mmHg) were evaluated. We included subjects with WHO group I and IV PH. According to the availability of data for all time points, 130 patients, aged 18 to 90 years, were included in the study. Clinical performance status, laboratory tests, echocardiography, capillary blood gas analysis, and pulmonary function testing at five different time points (baseline assessment and yearly follow-ups from 2015 to 2018) were analysed. The study inclusion process is depicted in Additional file 1: Fig S1.

### Ethics

All participants gave written informed consent for study participation, for use of their medical records and biological material. All samples and data were fully anonymized, the study was approved by the local ethics committee (Approval numbers: AM2544, 239/4.12 and 273/5.7, AN2017-0009369/4.15) and performed in accordance with the Declaration of Helsinki.

### Blood sampling and laboratory testing

Blood sampling was carried out via routine peripheral vein puncture and analysed by standardized ISO-certified procedures at the local laboratory. Blood gas analysis was obtained via punctuation of the hyper-perfused earlobe following Finalgon^®^ (Sanofi-Aventis, Germany) application.

### Risk assessment

The baseline risk for PH associated mortality was evaluated with seven different risk assessment strategies (for details refer to Additional file 1: Additional methods, Tables S1, S2). To compare REVEAL 2.0 and REVEAL Lite 2 to ERS/ESC risk scores, 3-categoric versions of the REVEAL tools were applied, as previously published [16, 17]. Follow-up risk stratification was performed using mRASP, FPHR3p, and refined versions of the FPHR3p model.

### Statistical analysis

Data were analysed with statistical analysis software package (IBM SPSS Statistics version 24.0, IBM, USA) and R (R Foundation for Statistical Computing). A detailed description of the used statistical methods is presented in Additional file 1: Additional methods section.

## Results

### Patients’ characteristics at baseline and follow-up

Patients with precapillary PH were evaluated at baseline (defined as the date of the first diagnostic RHC) and 4 consecutive yearly follow-ups (Table 1 and Additional file 1: Table S3). The majority of patients were categorized into WHO group I (78%), with IPAH being the most frequent diagnosis (67%). Eleven percent presented with connective tissue disease-associated PAH (CTD-PAH; WHO group I) and 22% were diagnosed with chronic thromboembolic PH (CTEPH, WHO group IV). At baseline, subjects mainly presented with WHOFc III (54%) and WHOFc II (33%), with a mean SMWD of 321 (± 132) meters. The observed 5-year mortality was 24%, and 43 individuals died throughout the study period. Following the diagnosis of precapillary PH, the majority of patients received specific medication. Accordingly, at follow-up, an overall significant improvement of various clinical, echocardiographic, functional, and laboratory parameters was observed (Table 1).

**Table 1:** Patients’ characteristics at baseline and last follow-up

	Baseline (N = 130) Mean ± SD	Last follow-up (N = 87) Mean ± SD	p-value Baseline to 2018
Age years	62 ± 15	67 ± 15	
Female no. (%)	78 (60)	55 (63)	
BMI kg/m	27 ± 6	26 ± 6	0.004
WHOFc I/II/III/IV no. (%)	5/43/70/12 (4/33/54/9)	21/30/33/3 (24/35/38/3)	< 0.001
SMWD m	321 ± 132	358 ± 143	0.005
Blood and serum parameters			
NT-proBNP ng/L	2631 ± 8747	1904 ± 7580	0.001
Haemoglobin g/L	140 ± 23	135 ± 26	0.962
Transferrin saturation %	22 ± 14	26 ± 29	0.448
Ferritin µg/L	126 ± 150	99 ± 89	0.106
RDW %	14.9 ± 1.9	14.9 ± 2.4	0.527
CRP mg/dL	0.9 ± 1.6	0.9 ± 1.8	0.959
GFR mL/min/1.73 m^2^	78 ± 28	75 ± 28	< 0.001
Creatinin mg/dL	1.1 ± 0.6	1.2 ± 1.1	0.003
Pulmonary function and BGA			
DLCO %	64 ± 23	65 ± 23	< 0.001
KCO %	82 ± 27	81 ± 27	0.002
paO_2_ mmHg	69 ± 14	67 ± 13	< 0.001
SO_2_%	93 ± 5	93 ± 5	0.001
Echocardiography			
RAA cm^2^	22 ± 5	18 ± 7	< 0.001
TAPSE mm	18 ± 4	23 ± 5	< 0.001
sPAP mmHg	61 ± 21	48 ± 21	< 0.001
Pericardial eff. no.(%)	21 (16)	5 (6)	0.038

Data are represented as mean ± 1 standard deviation (SD); N depicts the number of valid data for retrospective analysis. p-values depict Wilcoxon, Friedman (continuous variables) or Pearson Chi-Square (ordinal or dichotomous variables) test results for time-dependent changes during the observation period

BMI: body mass index, WHOFc: World Health Organization functional class, SMWD: six-minute walking distance, NT-pro BNP: N-terminal of the pro-hormone brain natriuretic peptide, RDW: red blood cell distribution width, CRP: C reactive protein, GFR: glomerular filtration rate, RAA: right atrial area, TAPSE: tricuspid annular plane systolic excursion, sPAP: systolic pulmonary arterial pressure, pericardial eff.: pericardial effusion; BGA, blood gas analysis; DLCO: diffusing capacity for carbon monoxide, depicted as percentage of normal, KCO: carbon monoxide transfer coefficient, also known as Krogh-Index (DLCO/VA), depicted as percentage of normal, paO2: arterial partial pressure of oxygen, SO_2_: oxygen saturation

### Risk assessment at baseline and follow-up

**Fig. 1:**
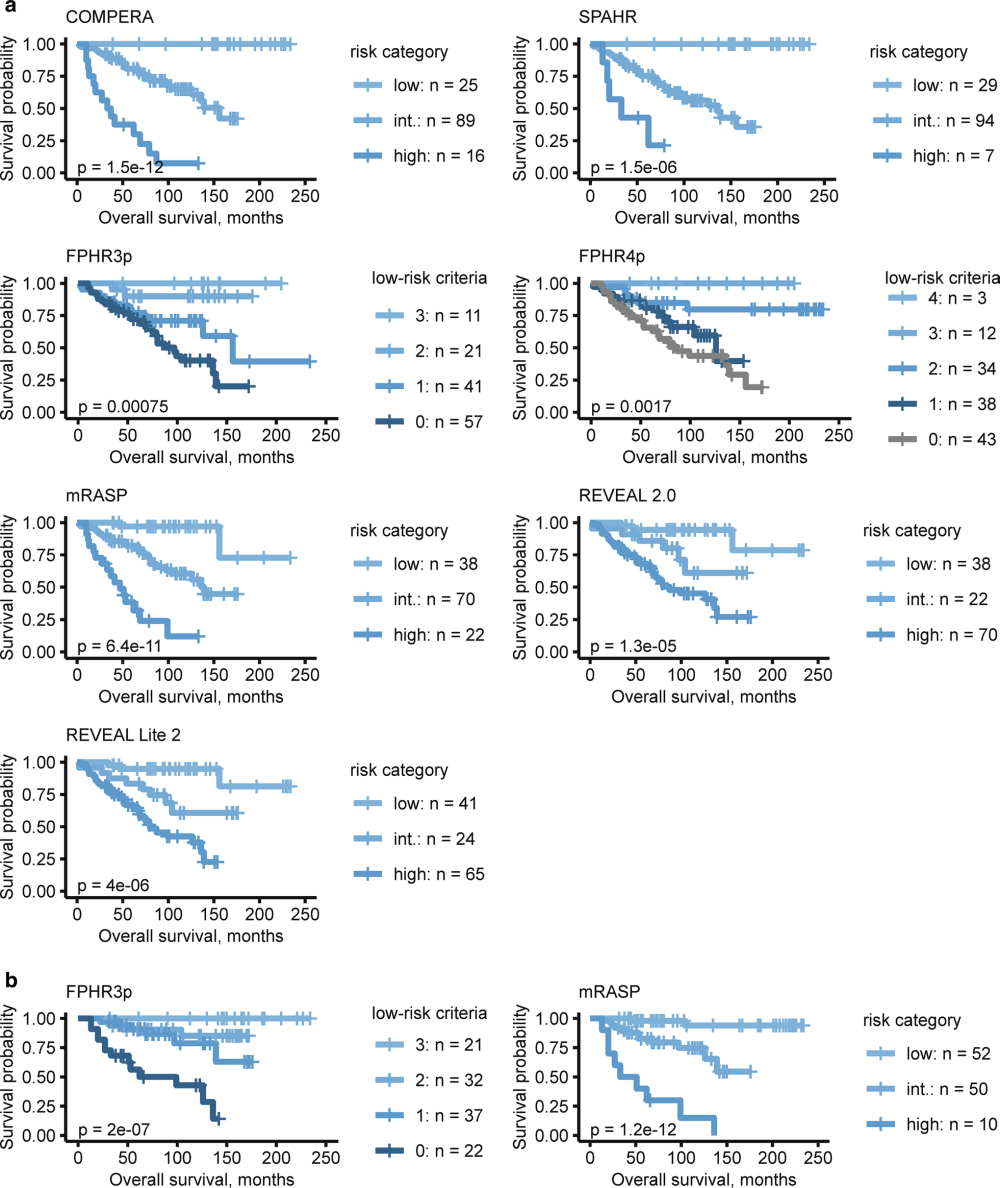
Observed mortality according to various risk assessment strategies. Mortality analysis by Kaplan–Meier (KM) modeling at baseline (**a**) and first follow-up (**b**). At baseline seven different risk-stratification approaches (COMPERA, SPAHR, FPHR3p, FPHR4p, mRASP, REVEAL 2.0 and REVEAL Lite 2) are depicted. At the first follow-up, KM modeling according to the FPHR3p and mRASP model is shown. The colour of KM curves is matched to score strata. Initial patient numbers in each stratum are presented for each score. Statistical significance was assessed with Wilcoxon test. For COMPERA, SPAHR, and mRASP three risk strata (low, int. = intermediate and high) are presented, for the FPHR models number of met low-risk criteria is shown

**Fig. 2:**
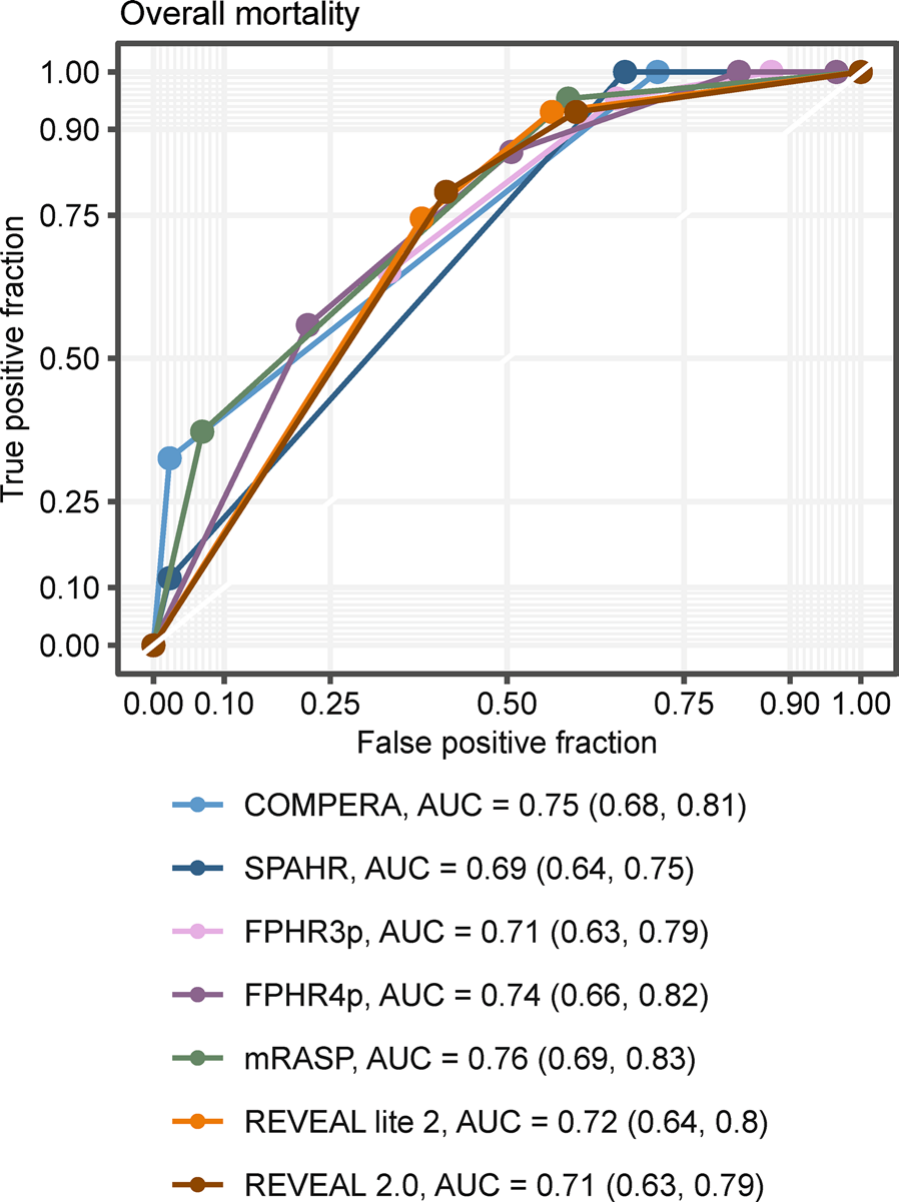
Mortality prediction performance of various risk assessment strategies at baseline. The sensitivity and specificity of various risk assessment tools were determined by ROC analysis according to baseline risk scores. As a response index overall mortality during the study period was used. At the bottom, curves are annotated with areas under the curve (AUC) and 95% confidence intervals (CI). A total of 130 precapillary PH patients were included in the analysis

We performed multi-parametric risk stratification applying the COMPERA, FPHR, SPAHR, REVEAL 2.0, REVEAL Lite 2, and mRASP risk-stratification tools [4, 7–9, 16, 17] (Fig. 1, Additional file 1: Tables S4 and S5). At baseline, the majority of patients fell into the intermediate-risk stratum (42 to 70% according to differential risk assessment tools, respectively), which would predict 1-year mortality of 5 to 10%. The observed 1-year mortality was 7%. When we assessed the correlation of the risk scales with survival within the study period using Cox proportional hazard models, the following C-index/AIC were obtained: 0.656/353 for FPHR3p, 0.670/355 for FPHR4p, 0.670/349 for SPHAR, 0.693/349 for REVEAL 2.0, 0.703/346 for REVEAL Lite 2, 0.724/335 for COMPERA, and 0.751/336 for mRASP. Thus, various risk assessment tools demonstrated a high predictive value, with a trend towards overestimation of mortality risk (Figs. 1, 2, and Additional file 1: Table S5). The latter was particularly true for models including RHC parameters, such as SPAHR, COMPERA, FPHR4p, and REVEAL 2.0, whereas models lacking RHC parameters, such as the mRASP and FPHR3p tended to predict a lower mortality risk (Fig. 1, Additional file 1: Tables S4, S5). Notably, when tested with receiver operating characteristic (ROC), risk scores including RHC parameters (namely COMPERA, SPAHR, FPHR4p and REVEAL 2.0) were not significantly superior for the prediction of mortality as compared to scores lacking RHC parameters (Fig. 2).

**Fig. 3:**
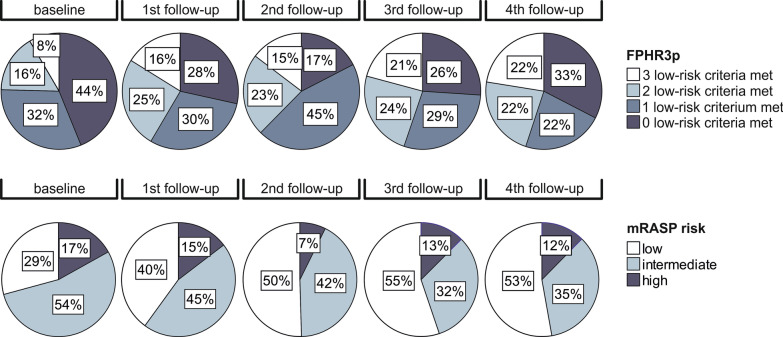
Mortality risk stratification according to the FPHR3p and mRASP risk assessment tools at baseline and follow-up. Risk assessment according to the three-parametric FPHR and mRASP risk stratification tools. The percentage of the total for each risk category is depicted. N_baseline_ = 130, N_1st follow-up_ = 112, N_2nd follow-up_ = 103, N_3rd follow-up_ = 93, N_4th follow-up_ = 87

As risk-assessment strategies excluding RHC parameters performed well at baseline, we tested the clinical usefulness and predictive value of the FPHR3p and mRASP at follow-up. In line with the aforementioned clinical and functional improvement following treatment initiation, patients shifted from higher to lower risk categories (Fig. 3). Notably, with both risk models, major improvements were mainly seen between baseline and the first follow-up, whereas at third and fourth follow-up risk strata distribution remained roughly unchanged.

### Parameter specific hazard analysis and refinement of risk stratification

**Fig. 4:**
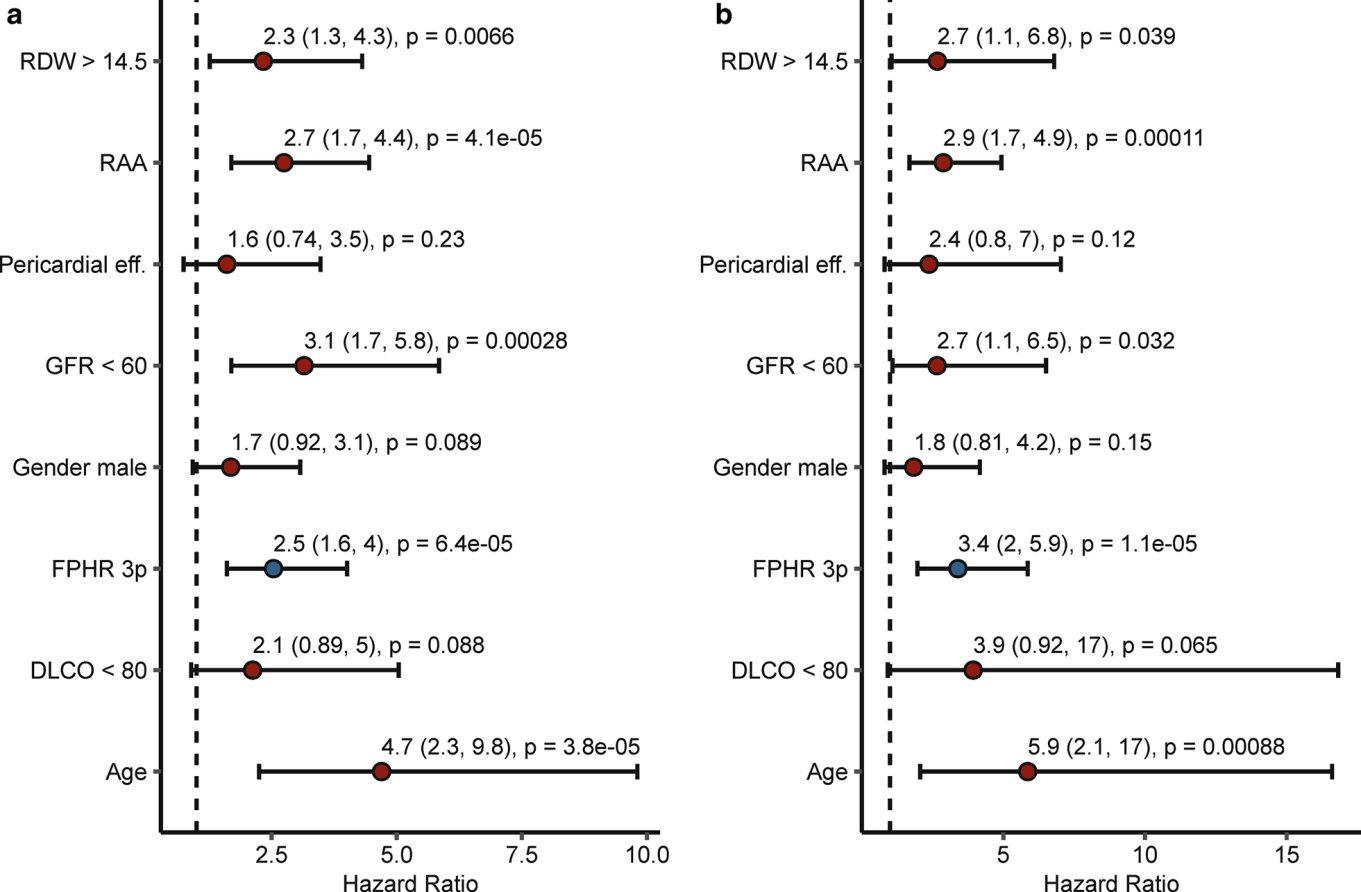
Univariate survival modeling for candidate FPHR3p-modifying variables. Candidate FPHR3p-score modifying variables were stratified and weighted, using the following cut-offs: age ≤ 40 y, 41–65 y, > 65 y; glomerular filtration rate (GFR, calculated by the MDRD-IDMS formula) ≤ 60 or > 60-mL/min/1.73 m^2^, right atrium area (RAA) < 18, 18–26, > 26 cm^2^, diffusion capacity for carbon monoxide (DLCO) < 80 or ≥ 80%. The prognostic value of each parameter for overall mortality risk prediction when assessed at baseline (**a**) and first follow-up (**b**) were evaluated by univariate Cox proportional hazard modeling. Points depict hazard ratios (HR), whiskers represent 95% confidence intervals (CI). Points are labeled with HR, 95% CI, and p values. Statistical significance (HR ≠ 1) was assessed with the Wald Z test. N_baseline_ = 130, N_1st follow-up_ = 112, N_deceased_ = 43

**Fig. 5:**
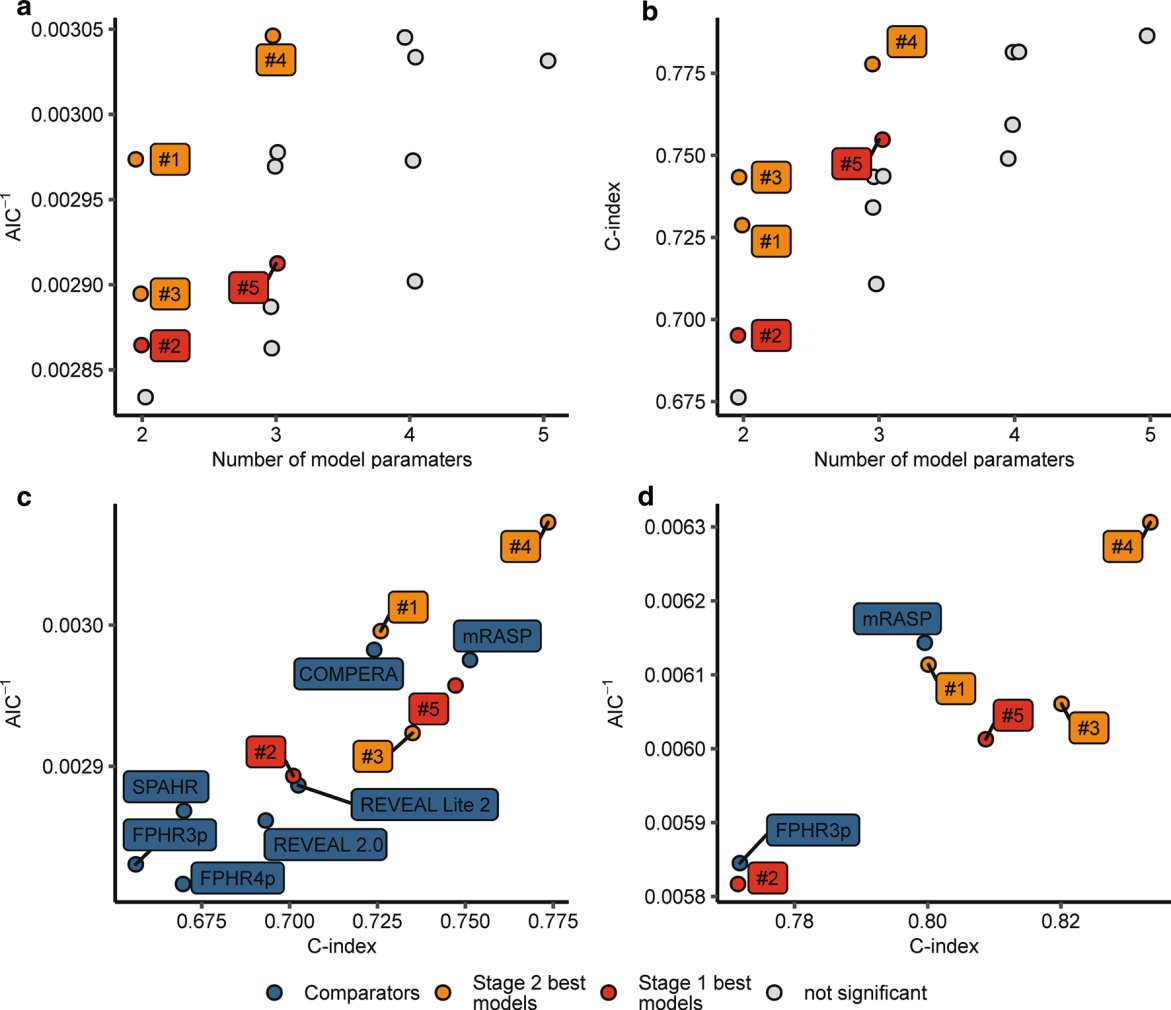
Refinement of the FPHR3p risk model and comparison to other mortality risk assessment strategies. Candidate variables to refine FPHR3p risk modeling were analysed in a step-wise model selection process as depicted in Additional file 1: Fig S2. In brief, 0–3 combinations of significant prognostic variables previously identified together with FPHR3p were correlated with overall survival at baseline (stage 1 model selection) and first follow-up (stage 2 model selection) using multivariate Cox proportional hazard models. At each stage, models with significant estimate sets (Wald Z-test), better survival fit (likelihood ratio test (LRT) vs. FPHR3p-alone model), and better prediction power (Akaike information criterion (AIC) and concordance index (C-index)) than the FPHR3p-alone model were selected. **a**, **b** Relationship between inverted AIC (**a**), C-index (**b**) and the number of model estimates for FPHR3p-modifying variable sets according to baseline patient risk parameters. Each point represents a single model, point colour codes for model performance in stage 1 and 2 selection steps. Stage 1 and 2 best models were labeled with names (#1 to #5). **c**, **d** Optimal modifying FPHR3p variables sets identified in previous analyses were used to calculate FPHR3p-derived scores (for used low risk-criteria cut-offs refer to this figure). The prediction power of modified FPHR3p models in comparison to other risk assessment strategies is shown according to the inverted AIC/C-index relationship for each studied model. Prediction power according to risk parameters obtained at baseline (**c**) and first follow-up (**d**) are depicted. Each point represents a single model, whereas best predicting models are found in the right upper corner. Only models with pLRT < 0.05 and regression estimate p’s < 0.05 are shown. Blue: values for comparator risk scales. Highlighted are: red—models with better performance than comparator risk scales, orange—top 10 best C-index models. Best performing test models are labelled with names and include following parameters: #1 FPHR3p + age, #2 FPHR3p + GFR, #3 FPHR3p + RAA, #4 FPHR3p + RAA + age, #5 FPHR3p + RAA + GFR. GFR = glomerular filtration rate, RAA = right atrium area, N_baseline_ = 130, N_1st follow-up_ = 112, N_deceased_ = 43

**Fig. 6:**
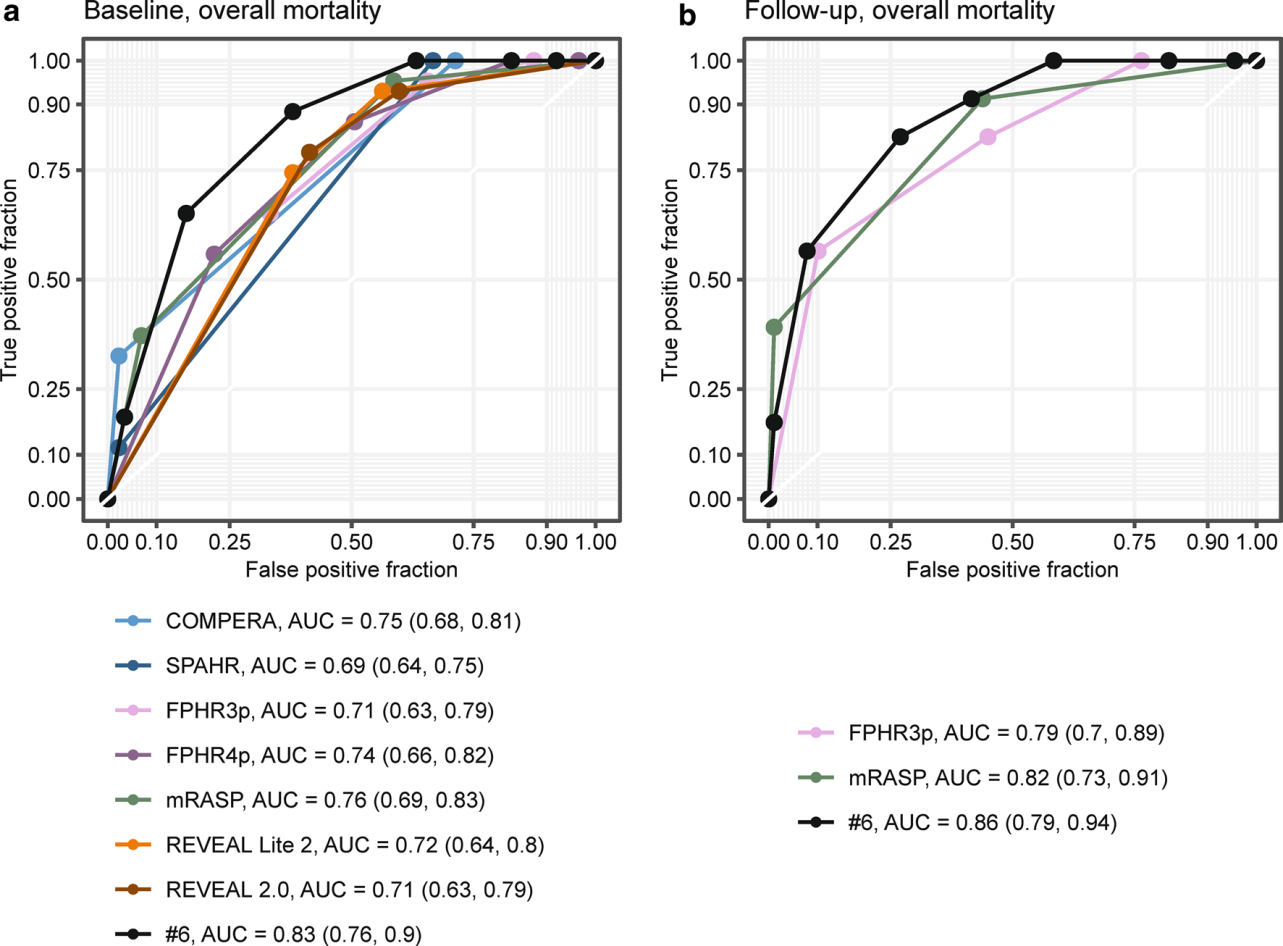
ROC analysis of the refined FPHR3p model in comparison to other risk assessment strategies. The sensitivity and specificity of various risk assessment tools were determined by ROC analysis according to baseline risk scores (**a**) or calculation of mortality risk at first follow-up (**b**). As a response index overall mortality during the study period was used. At the bottom, curves are annotated with areas under the curve (AUC) and 95% confidence intervals (CI). The best AUC value, which is reached by the refined FPHR3p model (test model = FPHR3p + age + RAA), is additionally presented in the plots. N_baseline_ = 130, N_1st follow-up_ = 112

**Fig. 7:**
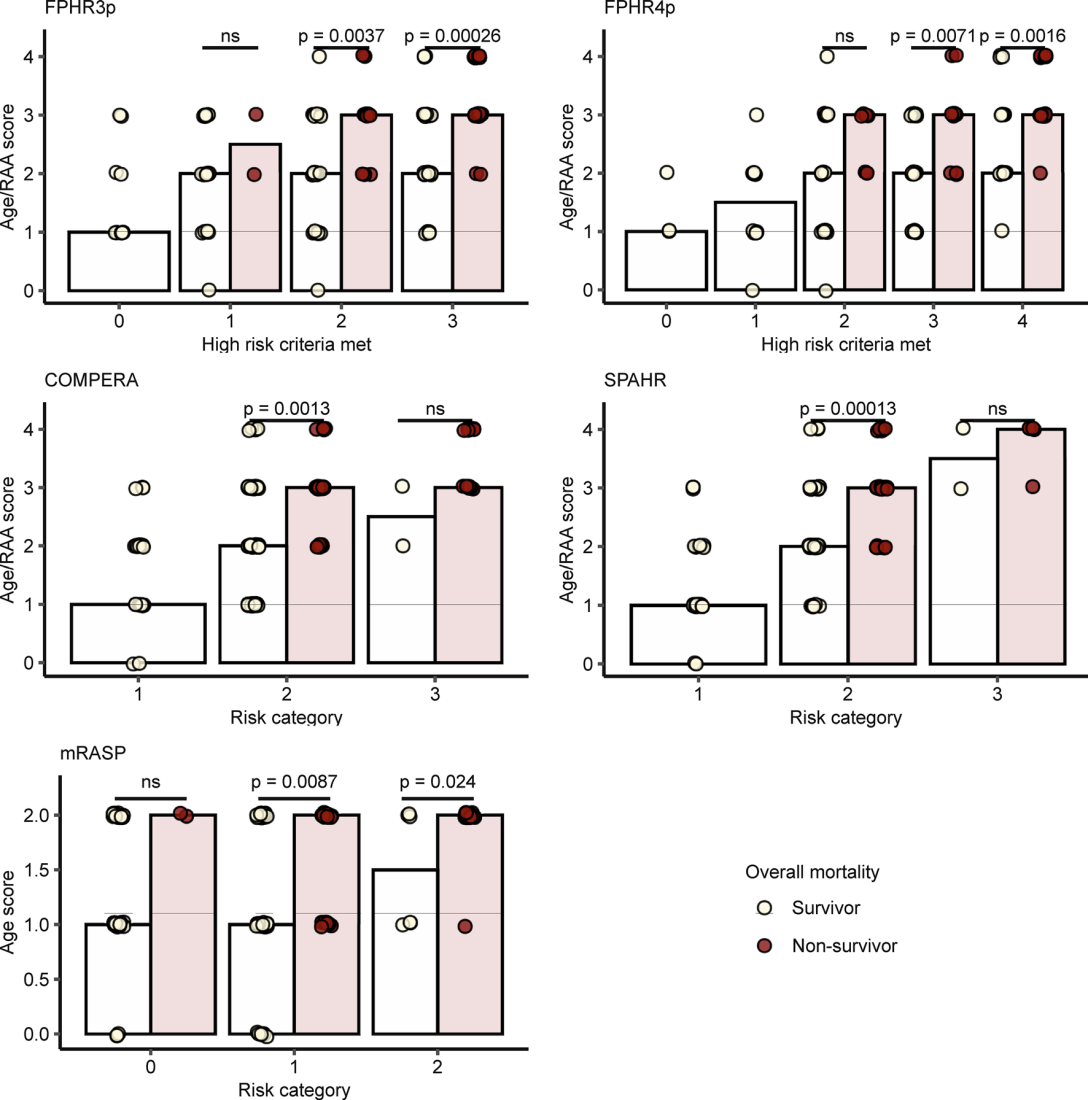
Age and right atrial area differentiate between PAH/CTEPH survivors and non-survivors classified as intermediate and high-risk individuals by established ERS/ESC PAH risk scores. PAH individuals at first consultation were stratified by the number of FPHR3 and FPHR4 high-risk criteria or SPAHR, COMPERA, and mRASP risk classes. Age score (0 for age ≤ 40, 1 for 40 < age ≤ 65, 2 for age > 65 years) and age/RAA score (sum of age score and 0 for RAA ≤ 18, 1 for 18 < RAA ≤ 26 and 2 for RAA > 26) was compared between PAH survivors and individuals who deceased during the observation period within each strata. For FPHR3, FPHR4, SPAHR, and COMPERA, age/RAA score values were compared, for mRASP, which already accounts for RAA, the age score was analyzed. Statistical significance for score differences between the survivors and non-survivors was determined by the Mann–Whitney U test, p values were corrected for multiple testing with the Benjamini–Hochberg method. Bars represent median age or age/RAA scores, points represent single study participants

Established risk-assessment tools performed well at defining very-low and low-risk patient populations but vastly overestimated mortality of the intermediate- and high-risk groups (Fig. 1 and Additional file 1: Table S5). This was particularly true for the FPHR3p score, which correctly identified low-risk individuals but lacked accuracy in other risk groups. Hence, we sought to find additional parameters, which may improve mortality prediction for the FPHR3p intermediate- and high-risk populations. First, we tested the correlation of various demographic and diagnostic non-RHC parameters, not included in FPHR3p, with overall survival, applying univariate Cox proportional hazard and Kaplan–Meier analyses (Fig. 4 and Additional file 1: Fig. S3). Next, we used random combinations of the best performing parameters derived from this analysis [namely age, glomerular filtration rate (GFR), right atrium area (RAA), red-cell distribution width (RDW), and diffusion capacity of carbon monoxide (DLCO)] to extend the original FPHR3p model and we tested the performance of such combined FPHR3p-based models for estimation of overall mortality in comparison to the original FPHR3p tool with multivariate Cox proportional hazard analyses (Figs. 5 and 6). By this approach, we found that the additional inclusion of age and RAA added the most accuracy to PH mortality risk prediction. The extension of the FPHR3p model with these parameters (FPHR3p-extended) resulted in a model outcompeting both, the FPHR3p and mRASP tools at baseline and first re-evaluation, as assessed by Cox proportional hazard (Fig. 5b, c) and ROC analysis (Fig. 6). In addition, the inclusion of stratified age and/or RAA enabled reliable differentiation between survivors and non-survivors among individuals classified as intermediate- and high-risk by the FPHR, COMPERA, SPHAR, and mRASP risk stratification tools (Fig. 7).

## Discussion

To date, the best method to predict the mortality risk of PH patients is still not defined, as current risk stratification strategies are controversially discussed regarding the inclusion of different risk factors [3–11, 14, 19]. Various study groups have compared currently available risk assessment tools for PH, and it has been reported that the “low-risk focused” FPHR model may be superior in the identification of PH patients with excellent long-term survival as compared to “score and average” models such as the COMPERA or SPAHR tool [20]. In this context, we herein compared three models using a “score and average” approach, namely COMPERA, SPAHR, and mRASP, and the “low-risk focused” approach by the FPHR risk assessment model. When applied at baseline, all these tools accurately predicted PH patients’ mortality during the observation period. Slight differences became apparent only on closer inspection. The most prominent discrepancy was seen when comparing tools including or lacking RHC parameters. Interestingly, risk assessment strategies including RHC tended to overestimate patients’ mortality risk as compared to models without RHC data. This assumption is strengthened by previous data, demonstrating that follow-up RHC does not improve the accuracy of risk prediction [21]. Additionally, risk assessment tools lacking RHC parameters, such as REVEAL Lite 2 or mRASP, were reported to have comparable performance to models including RHC parameters [8, 17, 22]. Consequently, RHC remains an obligatory tool for the establishment of the diagnosis of PH and is useful to guide treatment decisions, but it may not be an essential part of risk-stratification in PH patients.

Accuracy of risk prediction may benefit from a high number of included risk factors, as the highly comprehensive REVEAL 2.0 score was reported to be of superior predictive power as compared to the FPHR and COMPERA models [10]. Still, in the herein presented PAH/CTEPH cohort according to c-index calculation, the REVEAL 2.0 tool did not significantly outperform ERS/ESC based risk assessment tools but achieved a similar C-index compared to its establishment within the REVEAL cohort [16, 17]. The improved performance of ERS/ESC derived scores may be mainly explained by the different characteristics of the herein analysed PH cohort and the REVEAL registry patient cohort. First of all, we also included WHO group IV patients, whereas REVEAL solely focuses on WHO group I individuals. Secondly, patients included in the REVEAL analyses were significantly younger as compared to the herein presented cohort, and age demonstrated to be a major driver of mortality in the presented analysis.

Additionally, various risk factors included in the REVEAL 2.0 tool did not significantly improve risk estimation in our cohort, suggesting a redundant role of these factors for mortality risk prediction. This assumption is supported by current data from the REVEAL study group, demonstrating a comparable granularity of the REVEAL 2.0 (including 14 parameters) and the abridged REVEAL Lite 2 score, which only includes six non-invasive parameters [16]. Accordingly, Benza et al. reported that WHOFc, NT-proBNP, and SMWD were the most highly predictive parameters and that a REVEAL Lite 2 score including only these three parameters predicted mortality with a c-index of 0.72, whereas the six parametric REVEAL Lite 2 score achieved a c-index of 0.73 [17]. Notably, these parameters are also included in the mRASP score, which only includes four non-invasive parameters and demonstrated high predictive accuracy in our cohort. Contrarily, the FPHR3p, which also includes these three parameters, but lacks the evaluation of RAA, demonstrated a substantially lower prediction accuracy as compared with the mRASP model. Mechanistically, RAA has been shown to provide information on adverse ventricular remodeling, and this easily accessible parameter has been repeatedly proven of prognostic significance in PH [23, 24]. Thus our data suggest a significant role of imaging in PH mortality risk prediction and is supported by PH expert consensus statements [8, 9, 21, 25].

We herein demonstrate that currently used risk models lack accuracy in the distinction of intermediate-risk and high-risk PH patients. The latter is of high clinical relevance as the risk prediction significantly affects therapeutic decisions and scheduling of follow-up evaluations [4, 7, 9]. In this context, the application of the unweighted “score-and-average” ESC/ERS model is particularly challenging, as PH patients frequently demonstrate a mixed risk-factor profile, with some parameters within the high- and others in the intermediate- or low-risk range, and the ESC/ERS guidelines lack specific recommendations how to handle such cases [2]. Thus, we herein evaluated risk parameters for their potential to improve the characterization of intermediate- and high-risk PH patients. We demonstrate that adding combinations of known risk parameters, such as age and RAA, to the FPHR3p model significantly improves the accuracy of risk prediction in the intermediate and high-risk strata.

Additionally, evaluation of the age/RAA combination for patients classified as intermediate- and high-risk groups by ‘score and average’ tools such as COMPERA and SPHAR may also significantly improve the prediction of mortality. Notably, the inclusion of the combined age/sex parameter in the REVEAL 2.0 and REVEAL Lite 2 tools did not translate into their better performance than the other investigated risk stratification tools and the enhanced FPHR3p model. However, in our cohort, only age with two cutpoints at 40 and 65 years but not patient’s sex turned out to be a highly significant mortality predictor.

To conclude, our results suggest a step-wise risk modeling, with the use of simple scores for the screening of low-risk individuals, and extended scores for intermediate- or high-risk PH patients. This approach reduces the need for a generalized time- and resource-consuming risk assessment process for every patient, but rather focuses on the individual patients’ risk-status, and may help to use available resources effectively.

## Conclusion and perspective

Multiple accurate risk-assessment tools have been proposed for mortality prediction in PH. We herein present a comparative analysis of various currently available risk-assessment models and demonstrate their applicability in a cohort of precapillary PH patients. Although the predictive value of all tested models was good, we found some impreciseness in the intermediate- and high-risk strata, which is overcome upon inclusion of patient’s age and echocardiographic data.

## Supplementary Information

**Additional file 1: Fig. S1.** STROBE diagram for analysis cohort. **Fig. S2.** Model selection, score calculation, and score testing scheme for FPHR risk model refinement. **Fig. S3.** Kaplan–Meier analysis for candidate FPHR3p-modifying variables. **Table S1.** Variables used for risk stratification according to five different risk assessment tools. **Table S2.** Cut-offs used to define risk category for each risk parameters. **Table S3.** Baseline hemodynamic characteristics assessed by right-heart catheter at rest. **Table S4.** Mortality risk models including right-heart catheter parameters at baseline. **Table S5.** Mortality risk-assessment models according to the three-parametric FPHR and the mRASP model.

## Data Availability

All relevant data are available within the manuscript or supplementary section.
