# Supplementary material for: Risk assessment in precapillary pulmonary hypertension: a comparative analysis
Source: Respir Res. 2021 Jan 21;22:28. doi: 10.1186/s12931-021-01624-z (PMC7818775; doi:10.1186/s12931-021-01624-z)
Supplement: Supplementary file 1 — Additional file 1: Fig. S1. STROBE diagram for analysis cohort. Fig. S2. Model selection, score calculation, and score testing scheme for FPHR risk model refinement. Fig. S3. Kaplan–Meier analysis for candidate FPHR3p-modifying variables. Table S1. Variables used for risk stratification according to five different risk assessment tools. Table S2. Cut-offs used to define risk category for each risk parameters. Table S3. Baseline hemodynamic characteristics assessed by right-heart catheter at rest. Table S4. Mortality risk models including right-heart catheter parameters at baseline. Table S5. Mortality risk-assessment models according to the three-parametric FPHR and the mRASP model. [file 12931_2021_1624_MOESM1_ESM.docx]

**Supplementary material**

**Supplementary methods**

**Study population and design**

According to current clinical classification by the ESC/ERS guidelines, the majority of patients had WHO group 1 PH (78%), with mainly idiopathic PAH (iPAH) or connective tissue disease-associated precapillary PH (CTD-PH). Additionally, subjects with WHO group 4 inoperable chronic thromboembolic pulmonary hypertension (CTEPH)) (22%) were included. CTD-PH individuals suffered either from CREST (calcinosis cutis, Raynaud phenomenon, esophageal dysmotility, sclerodactyly, telangiectasia) syndrome, systemic sclerosis (SSc), systemic lupus erythematosus (SLE), or mixed connective tissue disease. Individuals with operable CTEPH, cardiac shunts, or haemolytic anaemias were not included in the study.

**Risk assessment**

We used the current ERS/ESC and REVEAL risk assessment tool to calculate a mean risk category for each patient, obtained by scoring for each category (1 for low-risk, 2 for intermediate-risk, and 3 for high-risk) followed by the summation of all scores and dividing by the number of included variables [1]. The ERS/ESC risk estimation was applied by utilizing abbreviated versions of the ERS/ESC risk assessment strategy, as evaluated in the COMPERA register and SPAHR [1, 2]. A risk assessment based on the achievement of defined low-risk criteria, as evaluated in the FPHR, was performed using either a four-parametric approach including RHC parameters or a three-parametric score using NT-proBNP instead of RHC parameters, as previously published [3]. Additionally, the modified Risk Assessment Score of PAH (mRASP), a risk assessment score not including RHC parameters, which was validated in comparison to the REVEAL risk equation, was implemented [4]. REVEAL scores were used in their 3-categoric form to allow comparison with ERS/ESC risk stratification.

Refinement of FPHR3p model was achieved by a step-wise approach (S2 Fig). First, candidate modifying variables (age, gender, GFR, presence of pericardial effusion, RAA, DLCO, and RDW) were tested for their correlation with overall survival at baseline and the first follow-up using univariate Cox proportional hazard models. Next, the variables proving significant at both time points (age, GFR, RAA, and RDW) were used for the generation of FPHR3p-derived models consisting of the FPHR3p score (0 – 3 coding for the number of missing low-risk criteria) and random combinations of 1 to 3 modifying parameters. Subsequently, such variable combinations were correlated with overall survival at baseline (stage 1 model selection) and first follow-up (stage 2 model selection) using Cox proportional hazard models. At each stage, models with significant estimate sets (Wald Z-test), better survival fit (likelihood ratio test (LRT) vs. FPHR3p-alone model), and better prediction power (Akaike information criterion (AIC) and concordance index (C-index)) than the FPHR3p-alone model were selected.

**Statistical analysis**

Following descriptive statistics including tests for homoscedasticity and data distribution, parametric or non-parametric tests were applied as appropriate. For group comparisons parametric Student`s t-test and repeated measures analysis of variance (ANOVA) or non-parametric Mann-Whitney-U test and Kruskal-Wallis test were applied, whereas trends over time analyses were performed with paired Student`s t-test, Wilcoxon test, or Nemar`s test, as appropriate.

The comparative analysis of various risk parameters and models was performed with univariate and multivariate Cox hazard analysis. The hazard ratio for overall mortality for each included parameter was tested with the Wald Z test. Prediction power for each risk model was assessed by the calculation of the Akaike information criterion (AIC) and the concordance index (C-index). Additionally, the specificity and sensitivity of each risk model were analysed with receiver operating characteristic (ROC).

**Supplementary Figures and Tables**

| Re-evaluation for each time-point or death during the observation period  n=153 |  |  |
| --- | --- | --- |
|  |  | Patients with missing data or no follow-up evaluation,  n=23 |
| Complete variables for all analysed parameters and time-points  n=130 |  |  |
|  |  |  |
| Analysis cohort, n=130 |  |  |

**S1 Fig. STROBE diagram for analysis cohort**


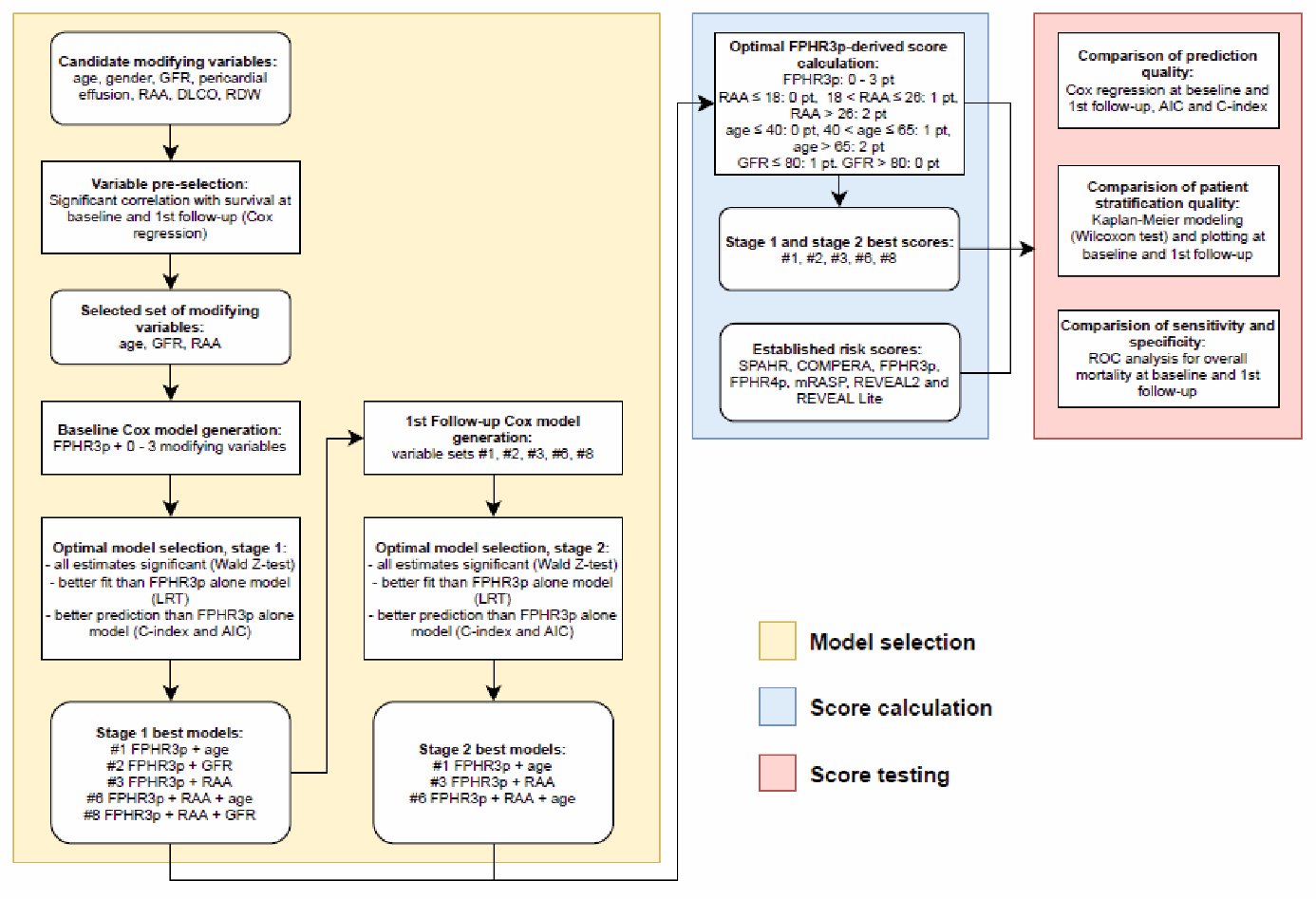


**S2 Fig. Model selection, score calculation, and score testing scheme for FPHR3p risk model refinement.**

**
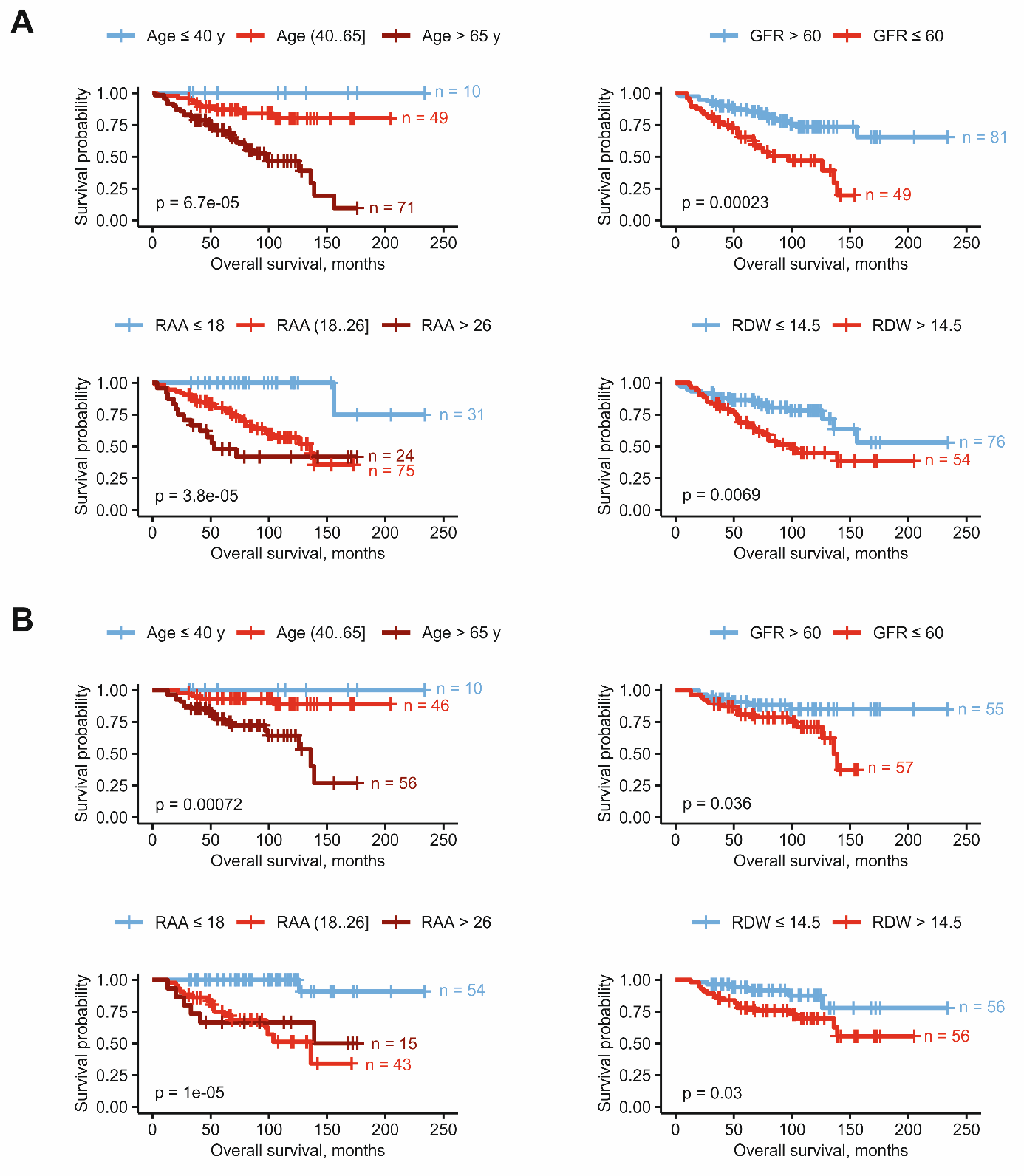
**

**S3 Fig. Kaplan-Meier analysis for candidate FPHR3p-modifying variables.**

Results of KM modeling for candidate variables identified as significant prognostic factors at baseline (A) and (B) first follow-up are shown. KM curves representing each variable strata are labeled with the initial number (n) of patients belonging to the particular strata. Statistical significance was assessed with the Wilcoxon test. Following cut-offs were used for stratification and weighting: age ≤40y, 41-65y, >65y; glomerular filtration rate (GFR, calculated by the MDRD-IDMS formula) ≤60 or >60-mL/min/1.73m^2^, right atrium area (RAA) <18, 18-26, >26 cm^2^, diffusion capacity for carbon monoxide (DLCO) <80 or ≥80%.

| **S1 Table. Variables used for risk stratification according to five different risk models** | |
| --- | --- |
|  | |
| **SPAHR (8 parameters)** | WHOFc, SMWD, NT-proBNP, RAA, pericardial effusion, RAP, CI, S_vO2_ |
| **COMPERA (6 parameters)** | WHOFc, SMWD, NT-proBNP, RAP, CI, S_vO2_ |
| **FPHR4p (4 parameters)** | WHOFc, SMWD, RAP, CI |
| **FPHR3p (3 parameters)** | WHOFc, SMWD, NT-proBNP |
| **mRASP (4 parameters)** | WHOFc, SMWD, NT-proBNP, RAA |
| **REVEAL 2.0 (14 parameters)** | PAH cause, sex, gender, renal insufficiency, WHOFc, all-cause hospitalization within previous 6 months, systolic blood pressure, heart rate, SMWD, NT-proBNP, pericardial effusion, diffusion capacity for carbon monoxide, RAP, PVR |
| **REVEAL Lite 2 (6 parameters)** | renal insufficiency, WHOFc, systolic blood pressure, heart rate, SMWD, NT-proBNP. |
| SPAHR: Swedish Pulmonary Arterial Hypertension Registry, FPHR: French Pulmonary Hypertension Registry, mRASP: modified Risk Assessment Score of PAH, WHOFc: World Health Organization functional class, SMWD: six-min walking distance, NT-proBNP: N-terminal of the pro-hormone brain natriuretic peptide, RAP: right atrial pressure, CI: cardiac index, RAA: right atrial area, S_VO2_: mixed venous oxygen saturation, PAH: pulmonary arterial hypertension, PVR: pulmonary vascular resistance. | |

| **S2 Table. Cut-offs used to define risk category for each risk parameter** | | | |
| --- | --- | --- | --- |
|  | | | |
| **Risk parameter** | **low** | **intermediate** | **high** |
| **WHOFc** | I/II | III | IV |
| **SMWD (m)** | <440 | 440-165 | <165 |
| **NT-proBNP (ng/L)** | <300 | 300-1400 | >1400 |
| **RAA (cm^2^)** | <18 | 18-26 | >26 |
| **Pericardial effusion** | no | no or minimal | yes |
| **RAP (mmHg)** | <8 | 8-14 | >14 |
| **CI (L/min/m^2^)** | ≥2.5 | 2.0-2.4 | <2.0 |
| **S_VO2_ (%)** | >65 | 65-60 | <60 |
| **FPHR low risk criteria** | WHOFc I/II, SMWD >440m, RAP<8mmHg, CI >2.5 L/min/m^2^, NTpro-BNP <300ng/L | | |
| WHOFc: World Health Organization functional class, SMWD: six-min walk distance, NT-proBNP: N-terminal of the pro-hormone brain natriuretic peptide, RAA: right atrial area, RAP: right atrial pressure, CI: cardiac index, S_VO2_: mixed venous oxygen saturation, FPHR: French Pulmonary Hypertension Registry. | | | |

| **S3 Table. Baseline hemodynamic characteristics assessed by right-heart catheterization at rest** | |
| --- | --- |
|  | **mean ± SD** |
| mRAP mmHg | 11 ± 5 |
| mPAP mmHg | 42 ± 15 |
| PVR dyn·s·cm^−5^ | 1120 ± 718 |
| CI L·min^−1^·m^−2^ | 2.4 ± 0.5 |
| S_VO2_ % | 66 ± 8 |
| Data are represented as mean ± 1 standard deviation (SD); N=130. Abbreviations: mRAP: mean right atrial pressure, mPAP: mean pulmonary arterial pressure, PCWP: pulmonary capillary wedge pressure, PVR: pulmonary vascular resistance, CI: cardiac index, S_VO2_: mixed venous oxygen saturation. | |

| **S4 Table. Mortality risk models including right-heart catheter parameters at baseline** | |
| --- | --- |
|  | |
|  | **N (%)** |
| **Swedish PAH Register (SPAHR) - 8 parameters** |  |
| Low (1-year mortality below 5%) | 29 (22) |
| Intermediate (1-year mortality 5-10%) | 94 (72) |
| High (1-year mortality above 10%) | 7 (5) |
| **COMPERA - 6 parameters** |  |
| Low (expected 1-year mortality below 5%) | 25 (19) |
| Intermediate (1-year mortality 5-10%) | 89 (69) |
| High (1-year mortality above 10%) | 16 (12) |
| **French Pulmonary Hypertension Registry (FPHR4p) - 4 parameters** | |
| 4 low-risk criteria met | 3 (2) |
| 3 low-risk criteria met | 12 (9) |
| 2 low-risk criteria met | 34 (26) |
| 1 low-risk criterium met | 38 (29) |
| 0 low-risk criteria met | 43 (33) |
| **REVEAL 2.0 (3 categories)** |  |
| Low (1-year mortality below 5%) | 38 (29) |
| Intermediate (1-year mortality 5-10%) | 22 (17) |
| High (1-year mortality above 10%) | 70 (54) |

| **S5 Table. Risk categorization at baseline and follow-up according to the three- parametric FPHR and the mRASP model** | | | |
| --- | --- | --- | --- |
|  |  | |  |
|  | **Risk category**  **N/%** | **Deaths during the observation period**  **N/%** | |
| **FPHR3p risk category at baseline** | | | |
| 3 low-risk criteria met | 11 (8) | 0 (0) | |
| 2 low-risk criteria met | 21 (16) | 2 (10) | |
| 1 low-risk criterion met | 41 (32) | 13 (32) | |
| 0 low-risk criteria met | 57 (44) | 28 (49) | |
|  |  |  | |
| **mean FPHR3p risk category during the study** | | | |
| 3 low-risk criteria met | 13 (10) | 0 (0) | |
| 2 low-risk criteria met | 27 (21) | 1 (4) | |
| 1 low-risk criterion met | 46 (35) | 10 (22) | |
| 0 low-risk criteria met | 44 (34) | 32 (73) | |
|  |  |  | |
| **mRASP risk category baseline** | | | |
| low | 38 (29) | 2 (5) | |
| intermediate | 70 (54) | 25 (36) | |
| high | 22 (17) | 16 (73) | |
|  |  |  | |
| **mRASP risk category during the study** | | | |
| low | 54 (41) | 1 (2) | |
| intermediate | 53 (41) | 22 (41) | |
| high | 23 (18) | 20 (87) | |
|  |  | |  |

**Supplementary references:**

1. Galie N, Humbert M, Vachiery JL, Gibbs S, Lang I, Torbicki A, Simonneau G, Peacock A, Vonk Noordegraaf A, Beghetti M, et al: 2015 ESC/ERS Guidelines for the Diagnosis and Treatment of Pulmonary Hypertension. *Rev Esp Cardiol (Engl Ed)* 2016, 69:177.

2. Hoeper MM, Kramer T, Pan Z, Eichstaedt CA, Spiesshoefer J, Benjamin N, Olsson KM, Meyer K, Vizza CD, Vonk-Noordegraaf A, et al: Mortality in pulmonary arterial hypertension: prediction by the 2015 European pulmonary hypertension guidelines risk stratification model. *Eur Respir J* 2017, 50.

3. Kovacs G, Berghold A, Scheidl S, Olschewski H: Pulmonary arterial pressure during rest and exercise in healthy subjects: a systematic review. *Eur Respir J* 2009, 34:888-894.

4. Simonneau G, Montani D, Celermajer DS, Denton CP, Gatzoulis MA, Krowka M, Williams PG, Souza R: Haemodynamic definitions and updated clinical classification of pulmonary hypertension. *Eur Respir J* 2019, 53.
